# Cohort study on clustering of lifestyle risk factors and understanding its association with stress on health and wellbeing among school teachers in Malaysia (CLUSTer) – a study protocol

**DOI:** 10.1186/1471-2458-14-611

**Published:** 2014-06-17

**Authors:** Foong Ming Moy, Victor Chee Wai Hoe, Noran Naqiah Hairi, Brian Buckley, Petra A Wark, David Koh, HB(as) Bueno-de-Mesquita, Awang M Bulgiba

**Affiliations:** 1 Julius Centre University of Malaya, Department of Social & Preventive Medicine, Faculty of Medicine, University of Malaya, Kuala Lumpur, Malaysia; 2 Centre for Occupational and Environmental Health-UM, Department of Social & Preventive Medicine, Faculty of Medicine, University of Malaya, Kuala Lumpur, Malaysia; 3 Department of General Practice, National University of Ireland, Galway, Ireland; 4 Department of Surgery, University of Manila, Manila, Philipines; 5 Global eHealth Unit, Department of Primary Care and Public Health, School of Public Health, Imperial College London, London, United Kingdom; 6 PAPRSB Institute of Health Sciences, Universiti Brunei Darussalam, Brunei, Brunei Darussalam; 7 Saw Swee Hock School of Public Health, National University of Singapore, Singapore, Singapore; 8 Department for Determinants of Chronic Diseases (DCD), National Institute for Public Health and the Environment, Bilthoven, The Netherlands; 9 Department of Gastroenterology and Hepatology, University Medical Centre, Utrecht, The Netherlands; 10 Department of Epidemiology and Biostatistics, The School of Public Health, Imperial College London, London, United Kingdom

**Keywords:** Teachers, Cohort study, Work related stress, Clustering of lifestyle risk factors

## Abstract

**Background:**

The study on *C*lustering of *L*ifestyle risk factors and *U*nderstanding its association with *S*tress on health and wellbeing among school *T*each*er*s in Malaysia (CLUSTer) is a prospective cohort study which aims to extensively study teachers in Malaysia with respect to clustering of lifestyle risk factors and stress, and subsequently, to follow-up the population for important health outcomes.

**Method/design:**

This study is being conducted in six states within Peninsular Malaysia. From each state, schools from each district are randomly selected and invited to participate in the study. Once the schools agree to participate, all teachers who fulfilled the inclusion criteria are invited to participate. Data collection includes a questionnaire survey and health assessment. Information collected in the questionnaire includes socio-demographic characteristics, participants’ medical history and family history of chronic diseases, teaching characteristics and burden, questions on smoking, alcohol consumption and physical activities (IPAQ); a food frequency questionnaire, the job content questionnaire (JCQ); depression, anxiety and stress scale (DASS21); health related quality of life (SF12-V2); Voice Handicap Index 10 on voice disorder, questions on chronic pain, sleep duration and obstetric history for female participants. Following blood drawn for predefined clinical tests, additional blood and urine specimens are collected and stored for future analysis. Active follow up of exposure and health outcomes will be carried out every two years via telephone or face to face contact. Data collection started in March 2013 and as of the end of March 2014 has been completed for four states: Kuala Lumpur, Selangor, Melaka and Penang. Approximately 6580 participants have been recruited. The first round of data collection and blood sampling is expected to be completed by the end of 2014 with an expected 10,000 participants recruited.

**Discussion:**

Our study will provide a good basis for exploring the clustering of lifestyle risk factors and stress and its association with major chronic medical conditions such as obesity, hypertension, impaired glucose tolerance, diabetes mellitus, coronary heart diseases, kidney failure and cancers among teachers.

## Background

Teachers are one of the largest occupational groups globally and this is also true in Malaysia [1]. Schools are reported to be a potentially stressful environment [2,3]: previous research in an urban setting in Malaysia determined the prevalence of stress among secondary school teachers were 20.2% [4].

Lifestyle risk factors such as smoking, overweight / obesity, alcohol use, physical inactivity and unhealthy diet are associated with chronic diseases such as diabetes mellitus, cardiovascular diseases and several types of cancers [5-7]. However, these risk factors are not randomly distributed across populations, but often occur in combination with each other [8]. The clustering of risk factors is associated with a higher risk of diseases than can be expected from the individual risk factors alone [9].

Data are emerging that stress is associated with risk of chronic disease. Work-related stress has been associated with risk of metabolic syndrome [10,11], coronary heart disease [12,13], acute myocardial infarction [14], and potentially type II diabetes [15][16]. Work related stress may also reinforce the association between metabolic risk factors [11] and renal dysfunction [17]. However, two studies on work-related stress and breast cancer did not find an association [18,19].

The association between work-related stress and risk of chronic disease might be partly mediated by lifestyle factors. A review based on 46 studies found a modest association between job-related stress and unhealthy behaviours and the strongest relationships were those with a combination of several unhealthy behaviours [20]. In a study on middle-aged white-collar workers from Britain, Finland and Japan, job strain and working overtime were mostly weak and inconsistently associated with unhealthy health behaviours and obesity [21]. Nonetheless, in a study in Korea, exposure to a cluster of unhealthy lifestyle factors was more common among individuals with mild or moderate stress than among those with lower stress levels [22].

This study will provide information on the interaction between work related stress and clustering of lifestyle risk factors on health and wellbeing, and will provide insights into the importance and direction of future preventive measures. This is also the first study conducted on the teachers’ health and wellbeing in Malaysia. After establishing the teachers’ cohort, we will follow up participants through time to provide stronger evidence on the associations between (changes in) work related stress, lifestyle risk factors and future health outcomes. The clustering of lifestyle risk factors and their associations with work related stress could help to identify groups of teachers that are at the greatest risk of developing chronic diseases.

The data from this research will be a useful guide for both clinical and health policy decision making. It will be used by public health specialists to design health promotion strategies to improve the health and wellbeing of teachers. These strategies can also be of importance for related occupational groups like office workers and universities academicians.

## Methods

### Study design

Prospective cohort study.

### Setting

Primary and secondary schools in Peninsular Malaysia.

### Objective(s) of the Study

#### General objective

To study teachers in Malaysia with respect to clustering of lifestyle risk factors and work related stress, and subsequently, to follow-up the population for important health outcomes.

#### Specific objectives

a. To describe the prevalence of exposures of interest, i.e. work related stress and individual and combinations of unhealthy lifestyle risk factors, among teachers in Malaysia, and its changes over time

b. To compare differences in work related stress across clusters of lifestyle risk factors.

c. To determine the baseline and subsequent occurrence of chronic conditions of interest, i.e., obesity, hypertension, impaired glucose tolerance (IGT), diabetes mellitus (DM), coronary heart diseases (CHD), stroke, cancers and kidney failure

d. To assess associations between baseline and repeated measures of work-related stress and lifestyle risk factors (life course) and risks of obesity, hypertension, IGT, DM, CHD, stroke, cancers and kidney failure

e. To examine associations between environmental risk factors (air pollution, greens and walkability) with risks of obesity, hypertension, IGT, DM, CHD, stroke, cancers and kidney failure

### Sampling methods

**Figure 1:**
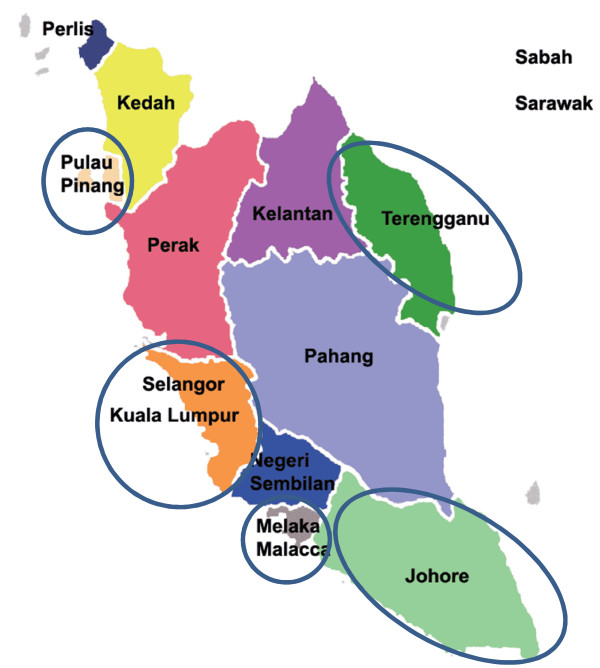
Selected states of Peninsular Malaysia for data collection.

A multi-stage sampling method was used. Out of 12 states in Peninsular Malaysia, six states (Figure 1 – Penang, Kuala Lumpur, Selangor, Melaka, Terengganu and Johor) were randomly selected. From each selected state, 70% of all public primary and secondary schools from every district are being invited for the study. Once a school agrees to participate, teachers who fulfil the inclusion criteria are invited to participate in the study. Participation is voluntary.

### Participants

All teachers on permanent employment of the selected public primary and secondary schools in the six selected states are eligible for inclusion. Only teachers with psychiatric illness are excluded. This information will be assessed from the questionnaire.

### Ethical considerations, patient information and written informed consent

Ethical approval was obtained (Reference Number: 950.1) from the Medical Ethics Committee of the University Malaya Medical Centre (UMMC) which governs all research involving human subjects in the Faculty of Medicine, University of Malaya. Approval was also granted by the Ministry of Education, Malaysia, each selected states’ Education Department and principals of all invited schools. All participants are briefed about the study and asked to provide written informed consent prior to data collection.

The informed consent form provides details on the background, objectives and procedures of the study. The participants will also be assured that all data remains confidential, identifiable information will only be available to those conducting field work, de-identified information will be used for both analysis and report writing, and that they have the right to withdraw from the study at any time.

### Sample size and its justification

Based on the statistics from the Ministry of Education, Malaysia for year 2013, there were a total of 412,042 teachers with 57% from primary and 43% from secondary schools. Males constituted about 30% of the total teacher population [1] . With the sampling of six out of 12 states and an estimated response rate of 25% of teachers from schools, a total of 10,000 teachers are expected to be recruited. The sample size of 10,000 was selected primarily for pragmatic budgetary and logistical reasons. However, it is considered to be large enough to detect NCD risk factor clustering in teachers exposed to work related stress. Previous research has suggested 20.2% of Malaysian teachers have work related stress. The 2005/6 Malaysian STEPS survey found that the percentage of the population having one, two, three, four or five risk factors for NCDs were 18.1%, 29.7%, 28.4%, 13.8% and 7.0% respectively [23]. For an observed prevalence of risk factor clustering in the whole teacher population of 30% with a precision of ±1% at confidence level of 95%, a minimum sample size of 8,067 would be required [24].

### Baseline and follow-up data and study endpoints

#### Baseline (Phase I) data collection

At baseline, all participants are required to complete the pretested and validated questionnaire (described below) and participate in the health assessment. The questionnaire includes socio-demographic characteristics, family history of chronic diseases and participants’ medical history; structured lifestyle questionnaire on smoking, alcohol consumption and physical activities (IPAQ) [25]; questions on fruits and vegetables consumption as well as types of oil and fat consumed, the job content questionnaire (JCQ) [26], depression, anxiety and stress scale (DASS21) [27], health related quality of life (SF12-V2) [28,29], the Voice Handicap Index 10 (VHI 10) [30] on voice disorder, and questions on chronic pain, sleep duration and obstetric history for female participants (Table 1). Some of these assessments will be repeated over time.

**Table 1:** Data collection

**Characteristics**	**Phase I (2013-2014)**	**Phase II (2015-2016)**
Socio-demographic	Date of birth	Spouse occupation
	Sex	Spouse education
	Ethnicity	
	Religion	
	Marital status	
Medical history	Diabetes mellitus	Birth weight
	Hypertension	Diabetes mellitus
	Heart disease	Hypertension
	Hypercholesterolemia	Heart disease
		Hypercholesterolemia
		Kidney failure
		Cancers
		Death and cause of death (cross check with National Death Registry)
Family history	Diabetes mellitus	Cancers (to specify)
	Hypertension	
	Cardiovascular disease	
	Hypercholesterolemia	
Occupation	Years of teaching	
	Highest qualification	
	Subjects taught	
	Duration of teaching	
	Duration for co-curriculum activities	
	Duration for administrative work	
Lifestyle factors	Physical activity (using International Physical Activity Questionnaire (IPAC) short form )	Physical activity (using International Physical Activity Questionnaire (IPAC) short form
	Smoking	Smoking
	Alcohol consumption	Alcohol consumption
	Diet :	Diet :
	Fruits & vegetable consumption Types of oil/fat consumed	Food Frequency Questionnaire
	Voice disorder ( Voice handicap index (VHI )10 questionnaire)	
	Duration of sleep	Duration of sleep
	Job content questionnaire	Job content questionnaire
Environmental exposure		Greens
		Walkability
		Fine particles
Acute outcomes	Depression, anxiety & stress	Depression, anxiety & stress
	( DASS 21)	( DASS 21)
	Pain	Pain
	Health related quality of life	Health related quality of life
	( SF12 v2)	( SF12 v2)
Obstetric history		Age of menarche
(for women only)		
	Parity	
	Life birth/still birth	
	History of Pre-eclampsia	
	History of gestational diabetes mellitus	
	Use of oral contraceptive pills	Use of oral contraceptive pills
	Menstrual status	Menstrual status
Anthropometry	Height	
	Weight	Weight
	Waist circumference	Waist circumference
	Hip circumference	Hip circumference
	Fat & muscle mass ( bioimpedance meter)	Fat & muscle mass ( bioimpedance meter)
Blood pressure	Systolic & diastolic	Systolic & diastolic
Clinical	Fasting blood glucose	Fasting blood glucose
	Full lipid profile	Full lipid profile
	Renal profile	Renal profile
Additional blood and urine prepared and frozen for further reference	Serum	Serum
		Heparin Plasma
		Buffy coat
		Spot Urine

#### Anthropometric assessment

Weight is measured in light clothing with shoes removed, to the nearest 0.1 kg using a digital calibrated floor scale (SECA 813, Hamburg, Germany). Height is measured without shoes to the nearest 0.1 cm with a portable stadiometer (SECA 217, Hamburg, Germany). BMI is calculated in kg/m^2^. Waist circumference is measured to the nearest 0.1 cm at the umbilicus, between the tenth rib and the iliac crest using a flexible tape measure (SECA 203, Hamburg, Germany). Hip circumference is measured using the same tape measure at the widest portion of the buttocks. The cut-off used to identify abdominal obesity among Malaysian were according to the Asian standards [31] where waist circumference of 90 cm and 80 cm are used for males and females respectively. Fat mass and muscle mass are measured using the Bioelectrical impedance meter (TANITA, TBF-300A Body Composition Analyzer). Participants are required to remove shoes and socks before stepping on the machine.

#### Clinical health assessment

Clinical health assessment includes systolic and diastolic blood pressure, fasting blood glucose, fasting lipid profile and renal function test. Participants’ systolic and diastolic blood pressure are measured once on the left arm in a sitting position using a validated oscillometric blood pressure monitor (Omron HEM 907, Japan) [32]. Participants are required to rest for 5-10 minutes before taking the measurement.

All biochemical analyses (fasting blood glucose, fasting lipid profile and renal function test) are conducted by the Clinical Diagnostic Laboratory of UMMC. Fasting blood glucose, fasting lipid profile and renal function tests are analysed using the Dimension® clinical chemistry system which is an in-vitro diagnostic test. LDL cholesterol is calculated using the Friedewald formula [33]: (LDL cholesterol) = (total cholesterol) – (HDL-cholesterol) – (triglyceride)/5.

#### Collection of biospecimen

Blood samples and urine samples will be collected at baseline and follow-up visits. From each participant, at baseline, four tubes of blood with an estimated total volume of 15 ml are collected. Out of these, two tubes (BD Vacutainer with Sodium Floride and Vacutainer SST II Advance) of 1.5 to 2.5 ml (Sample A) will be used for baseline measurements (health assessment) of blood lipids, fasting blood glucose and renal function test (mentioned in section on Clinical Health Assessment above). Another two tubes without anticoagulant of 5 ml whole blood (Sample B) are used for long term storage. All samples are kept protected against sunlight (as some biomarkers eg: vitamin E is light sensitive). The samples A and B are temporarily stored at 4 degrees Celsius in a cool box immediately after phlebotomy to preserve levels of markers sensitive to degradation due to higher temperatures. Samples A & B are processed in the field laboratories in the states. Samples A are spinned and stored as serum for health assessment while all samples B are spinned into approximately 5 ml of serum and subsequently divided in 10 aliquots of 0.5 ml of serum. These aliquots are stored at -80 or -20 degrees Celsius at the field laboratories until they are transported to University of Malaya (UM) biobank. We will also ensure that the entire cold chain from the time of drawing to storage at -80 or -20 degree Celsius should be not more than 4 hours. If only -20 degrees Celsius freezers are available in the field, the frozen serum will be transported back to the UM biobank within a week and is stored at -80 degrees Celsius.

#### Follow up (Phase II) data collection

Information such as birth weight, spouse’s education and occupation status which are not captured in Phase I will be enquired in Phase II data collection. Food frequency questionnaire and measurement environmental risk factors will also be administered in Phase II (described below). Additional blood sampling (serum, heparin plasma) and spot urine will be collected in Phase II too.

#### Food frequency questionnaire (FFQ)

A FFQ has been specifically developed for this study. Based on dietary data from 5409 respondents from all ethnic groups from the Peninsular Malaysia participating in the Malaysian Adult Nutrition Survey (MANS) [34], a food list was constructed based on the database approach by Block [35]. Similar food items were grouped into same food groups. Food items that contributed at least 90% of energy and nutrients intake were included in the food list. These food items were grouped into 17 food groups based on their nutrient contents. The FFQ asks for the usual dietary intake over the past year. The FFQ was then reviewed by four dietitians, pretested among students and staff of the university and revisions were made based on feedback received.

The FFQ will be validated among school teachers and clerical staff of schools in Kuala Lumpur. First, a total of 600 participants will be requested to administer the FFQ, after a gap of two weeks, the participants will be requested to fill a 7-day food record dairy. A subset (150) of the participants will be asked to provide a single 24 hour urine specimen for measuring urinary sodium, potassium and nitrogen to provide well-established objective measures for validation of the FFQ. All the 600 participants will be required to fill the FFQ again after three months from the first FFQ administration. The validated FFQ will be self-administered by all participants of CLUSTer. The results of the validation and reproducibility study will include further details about the FFQ and will be reported in a separate publication.

#### Measurement of environmental risk factors

The environmental risk factors selected are air pollution, walkability and greens. Air pollution will be assessed by monitoring the Air Pollution Index of the participants’ residential areas, while walkability and greens will be assessed using the objective walkability index using the geographic information systems (GIS) methodology established by Frank et al [36] and adapted by Leslie et al [37].

#### Additional blood sampling (plasma and buffy coat)

A second round of blood sampling will be conducted in Phase II to control for errors arising from single sampling. A larger volume of blood (20 ml) will be collected. It will be divided into two tubes of 7.5 ml whole blood; one for serum and one for heparin plasma and the preparation of buffy coats and two tubes of 1.5-2.5 ml samples (same as sample A) for repeat measurement of blood lipids, fasting glucose and renal function tests. Knowledge of the within-subject variation over a two year period of marker levels is important to allow for de-attenuation of risk estimates obtained in disease models. Thus, at the third round (if there is availability of funds), a second 7.5 ml heparin plasma sample will be included, as no plasma is collected at the first round. All samples will be divided in multiple aliquots, frozen and stored in a centralised biobank in the Faculty of Medicine, University of Malaya.

#### Spot urine

A spot urine specimen of 30 ml will be collected in Phase II. The participants need to wash his/her hand prior to voiding. There should be no touching of the inside of the specimen cup, including the lid. The participant should pass a small amount of urine out before collecting their sample. The collected urine of 30 ml per participant is divided into 6 x 5 ml of aliquots and are stored at -20 degrees Celsius for future analysis in a centralised biobank in the Faculty of Medicine, University of Malaya.

### Centralised biobank

An important part of the CLUSTer study is creating a centralised biobank. At the centralised biobank, all aliquots will be kept anonymously, with only codes for identification. All entries and usage of aliquots will be recorded. An estimation of 10 freezers will be needed for the storage of all samples. All freezers will be installed with alarm system and backup generator in the case of electricity disruption. An additional empty freezer will be available in case of breakdown of any of the ten freezers. These blood samples (serum, plasma and buffy coat) and urine samples are collected for future biomarker testing .

#### Follow-up for health status

In the absence of national registries for chronic diseases, active follow-up of health status will be carried out every two years using multiple sources including contacting the participants via telephone or face-to-face contact. Health events such as hypertension, diabetes mellitus, coronary heart diseases, stroke, cancers and kidney failure will be self-reported as doctors’ diagnosis. Death and cause of death will be obtained by linkage with the National Death Registry. Exposure status at subsequent rounds will be assessed similarly within the same season.

#### Dissemination of results to participants

Results on full lipid profile, fasting blood glucose and renal profile are disseminated to the participants. They will be referred to the nearest government clinics for further management if their results are beyond clinical reference ranges and thus may require medical attention.

#### Data entry

Data entry of questionnaires is conducted via two methods. Questionnaires used from March 2013 to February 2014 were entered manually while questionnaires from March 2014 onwards were designed using the TeleForm software [38]. TeleForm software offers flexibility in automatically classifying paper forms and converting information from paper into usable digital data. This will reduce time for data entry and errors arising from manual data entry.

### Quality assurance and quality control

The method of sampling, questionnaire design, physical examination, laboratory examinations and data management are standardised. Training of field staff involved in data collection, and staff handling data entry, checking, and cleaning will be implemented regularly. Data entry for the first four states will be conducted manually while all subsequent questionnaires will be scanned using the specialised software (TeleForm) and data extracted automatically. Computer programs are developed to check the logic and reasonable range of responses throughout the questionnaire to identify contradictory responses. Inconsistent records will be manually checked and corrected. Site visits by the investigators will be carried out regularly to ensure field staff carries out all procedures as per protocol. The quality and completeness of exposure and health outcomes follow-up data will be checked regularly.

To protect the confidentiality of participants, all clinical data, questionnaire data, laboratory data and biobank information will be de-identified. Participants’ names and address are kept separately in a master file where access is only available for authorised research staff. All data will be entered in a secured platform. Before the data are uploaded, data checks are performed to investigate completeness and correctness of the data. Furthermore, electronic data will be password protected, stored on the University of Malaya server and will only be accessible to the authorised research staff.

### Data analysis

Descriptive statistics will be presented as proportions, means ± standard deviations, or medians and interquartile ranges. Clustering of lifestyle risk factors will be examined using joint and pattern analyses (exploratory and score-based) [39]. The resulting patterns will be investigated for association with levels of stress using ANOVA and other regression techniques. Cox proportional hazard regression models will be fitted to estimate hazard ratios with 95% confidence intervals to assess the associations between baseline characteristics and subsequent risk of chronic disease. For repeated measures of exposure time-dependent models will be used. All regression models will be adjusted for established and potential confounders. The significance level will be pre-set at 0.05.

### Preliminary results

Data collection started in March 2013. As of the end of March 2014, data collection has been completed for four states, i.e., Kuala Lumpur, Selangor, Melaka and Penang. Approximately 6580 participants have been recruited. Currently these data are being entered and cleaned. The first round of data collection and blood sampling is expected to be completed by the end of 2014.

## Discussion

The main aim of the CLUSTer cohort is to study the cross-sectional and prospective associations between (clusters of) lifestyle risk factors including stress and risk of obesity, hypertension, impaired glucose tolerance (IGT), diabetes mellitus (DM), coronary heart diseases, kidney failure and cancers among teachers. To evaluate changes over time, repeat measures of exposure and enquiry of health outcomes will be carried out every two years. We are also interested in determining the contribution of gene-environment interactions to the above.

The CLUSTer cohort is one of only a few cohort studies in Malaysia. To our knowledge, there is only one other cohort study (The Malaysian Cohort) [40] in the country. The Malaysian cohort is a community cohort, while CLUSTer is an occupational cohort that is made up of a representative sample of public primary and secondary school teachers. The majority of studies in school have been on students rather than on the health and wellbeing of teachers. As teachers in the public sector are a “captive population” that can be traced easily through the Ministry of Education’s records if they are still employed or through pension records when they have retired, we predict a relatively low attrition rate.

Our multistage sampling method ensures recruitment of teachers from both urban and rural settings. Lifestyle and environmental exposure in these two settings may be different. This will enable us to better study these factors and its relationship with health.

The questionnaire used from March 2014 onwards is formatted according to scanning software (TeleForm) which will greatly reduce the time and errors in data entry. In addition, all blood samples are transported back to the central laboratory in UMMC for standardised methods of biochemical analyses eliminating most measurement bias in lab assays. Furthermore, a centralised biobank will be set up for more cost effective research where analyses of markers of exposure or of early disease in prediagnostic blood samples will only be carried out after an event has occurred using a cohort-nested case-control design or case-subcohort approach to minimise assay drift.

The second blood samplings in Phase II and III will enable us to identify the within-subject variation over a two year period of marker levels among the same participant. This is of utmost important to allow for de-attenuation of risk estimates obtained in disease models.

Some challenges have been encountered during the earlier phase of data collection. The teachers’ availability is dictated by school term and exam schedule. The duration of data collection in schools is less than nine months per year. Therefore, the planning on data collection schedule needs to be strictly followed according to the school calendar. In addition, as the research team needs to visit some schools that are more remote than the others, preserving the cold chain from the time of drawing of blood samples to storage at -80 degree Celsius to be as short as possible is a real challenge.

Due to the voluntary participation of cohort members, there may be an under-representation of those with lower health awareness although in preliminary analyses both participants and non-participants have a similar educational status. Comparisons between participants and non-participants at inclusion and during the follow up through the “non-participants cohort” should allow assessment of potential biases due to selection effects, but lack of sufficient information might be a problem. A healthy volunteer effect often tapers off with longer follow-up. Therefore, in the first years of follow-up, power might not be sufficient to detect weak associations.

In summary, the CLUSTer study will provide a good basis for exploring the clustering of lifestyle risk factors and its association with obesity, hypertension, impaired glucose tolerance (IGT), diabetes mellitus (DM), coronary heart diseases, kidney failure and cancers among teachers. There is also a distinct possibility of future collaboration with the Asian Cohort Study Consortium [41].

## Competing interest

The authors declare that they have no competing interests.

## Authors’ contributions

FMM conceived and designed the study, drafted and coordinated the manuscript. VCWH, NNH, BB, PAW, DK, HBBM, AMB participated in the design of the study and helped to draft the manuscript. All authors read and approved the final manuscript.

## Pre-publication history

The pre-publication history for this paper can be accessed here:

http://www.biomedcentral.com/1471-2458/14/611/prepub
